# SIRT2 puts the brakes on human β cell proliferation: therapeutic opportunities and next challenges

**DOI:** 10.1172/JCI197142

**Published:** 2025-10-01

**Authors:** Liora S. Katz, Donald K. Scott, Andrew F. Stewart

**Affiliations:** The Diabetes Obesity Metabolism Institute, The Icahn School of Medicine at Mount Sinai, New York, New York, USA.

## Abstract

The numbers of insulin-producing β cells in the pancreas are reduced in people with type 1 or type 2 diabetes, prompting efforts to replace these missing or lost β cells through transplant or regenerative medicine approaches. In this issue of the *JCI*, Wortham et al. describe a function for the deacetylase enzyme sirtuin 2 (SIRT2) in a novel pathway that acts as a brake on β cell proliferation. They show that inhibiting SIRT2 through pharmacologic or genetic approaches can induce human and mouse β cells to reenter a proliferative cell cycle. A surprising observation that remains unexplained is that the main targets of SIRT2 are mitochondrial oxidative phosphorylation (OxPhos) enzymes. It also remains unknown if and how these unanticipated acetylated OxPhos targets lead to cell-cycle entry. SIRT2 inhibitors will be a welcome addition to the growing repertoire of human β cell–regenerative drugs.

## Regenerative approaches to β cell loss in diabetes

Type 1 diabetes (T1D) and type 2 diabetes (T2D) incidence has reached epidemic proportions across the globe. T1D is driven by autoimmune destruction of β cells, and the residual insulin secretion by remaining cells is inadequate to control blood glucose levels. Although insulin resistance in the liver, skeletal muscle, and adipose tissue contributes to the pathophysiology of T2D, the mass, number, and function of insulin-producing β cells are also reduced in established T2D as compared with age-, BMI-, and sex-matched controls. The quest for a therapeutic advance that can restore the diminished mass and number of insulin-producing β cells in people with diabetes has been a long and challenging one, marked by promising advances and persistent hurdles. A new study by Wortham et al. in this issue of the *JCI* (1) introduces a potentially powerful strategy to this critical field of research.

Attempts to increase the mass and functional capacity of β cells in humans with T1D or T2D can be divided into two broad approaches. The first approach involves β cell replacement, exemplified by whole pancreas transplantation, usually in coordination with a kidney transplant, in a person with diabetes and kidney failure (2), as well as by transplantation of isolated human pancreatic islets (3, 4) and by transplantation of human embryonic stem cell–derived (hESC-derived) islets (5, 6). Each of these approaches has achieved success in humans but their use is limited by cost and/or the availability of human organ donor pancreata. These limitations have motivated a second type of approach: the development of drugs that could induce human β cells to regenerate in situ in the pancreas of humans with T1D and T2D. This type of approach encompasses a long list of strategies, including inhibitors of the kinase DYRK1A with small molecules like harmine (7); manipulation of TGF-β signaling with TGF-β inhibitors and BMPs (8), inhibitors of proteases such as serpinB1 (9); inhibitors of the β cell epigenetic regulator, menin (10); and β cell mitogenic proteins, such as glucagon-like peptide 1 (GLP1), gastrin, EGF, islet neogenesis-associated protein, a fragment of one of the Reg family proteins (INGAP), and R-spondin (11, 12). Several of these strategies are in various stages of development and could offer scalable, affordable alternatives to organ or tissue replacement. But none is perfect so far: there is room for novel approaches.

## SIRT2 links glycemia to β cell regeneration

**Figure 1:**
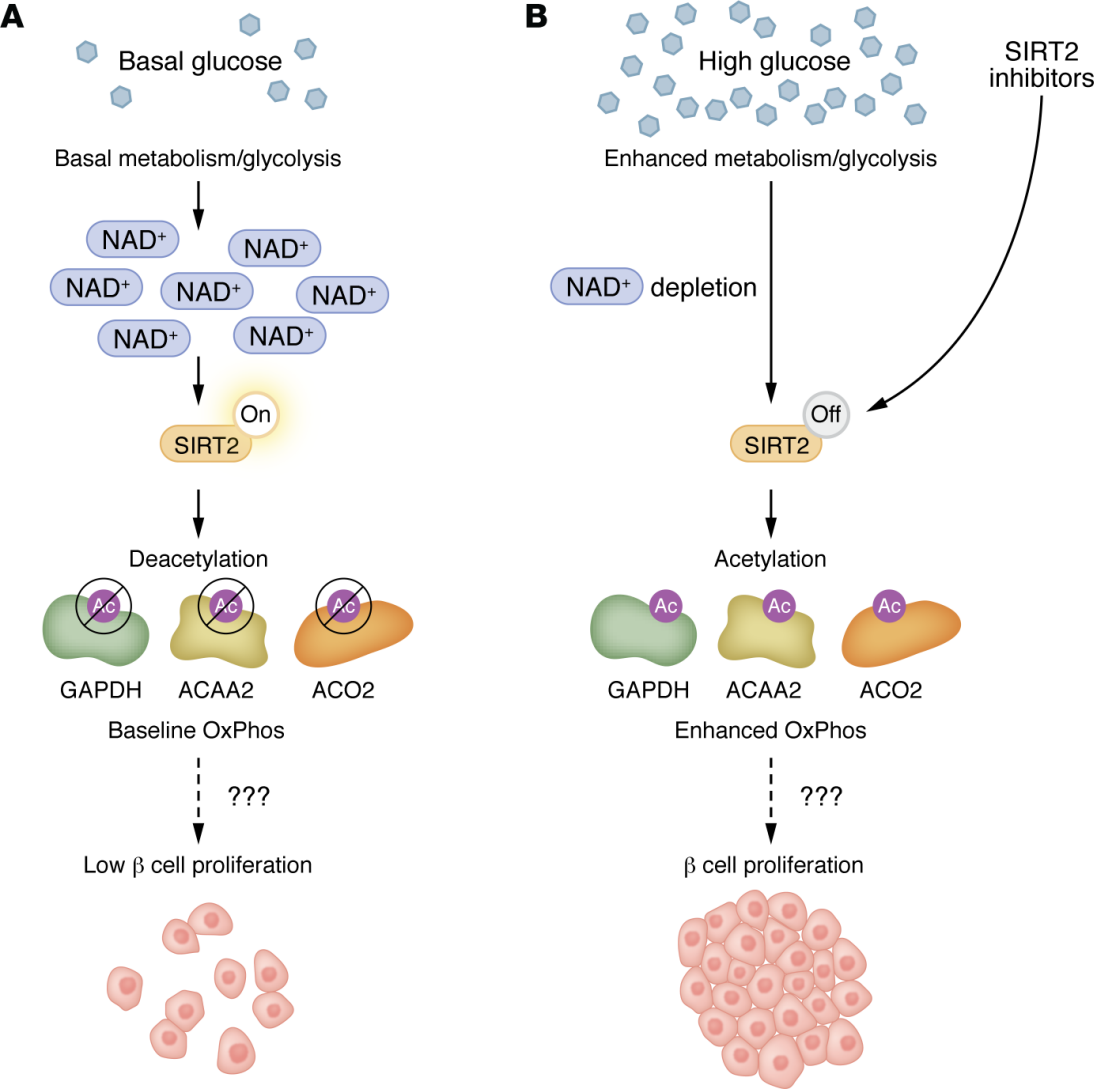
A glucose-driven, SIRT2-dependent metabolic switch regulates β cell proliferation. (**A**) Under basal glucose conditions, high NAD^+^ levels maintain active SIRT2 deacetylase activity. SIRT2 targets and deacetylates genes encoding multiple metabolic enzymes that support baseline glycolytic and OxPhos activity, including glycolytic enzymes (e.g., GAPDH and PKM), fatty acid β oxidation enzymes (e.g., ACAA2 and HADH), and TCA enzymes (e.g., ACO2 and SDHA), a milieu that is associated with low β cell proliferation. (**B**) In contrast, elevated glucose or pharmacological inhibition of SIRT2 increases glycolytic flux, elevates NADH, and depletes NAD^+^, leading to SIRT2 inhibition. This results in hyperacetylation of metabolic enzymes and enhanced metabolic flux through glycolysis and OxPhos and promotes adaptive β cell proliferation. Systemically in mice, SIRT2 inhibitors increase the potential for acetylation, activation of metabolism, and adaptive proliferation without losing the braking effect of a return to low glucose and the restoration of deacetylating SIRT2 activity. Ac, acetylation; PKM, pyruvate kinase M1/2; ACAA2, acetyl-coenzyme A acyltransferase 2; HADH, hydroxyacyl coenzyme A dehydrogenase; ACO2, aconitase 2; SDHA, succinate dehydrogenase subunit A.

Against this background, Wortham et al. have nominated small-molecule inhibitors of the sirtuin family member sirtuin 2 (SIRT2) as an innovative class of potential β cell–regenerative drugs for diabetes (Figure 1). SIRT2 is a deacetylase that requires NAD^+^ as a cofactor to remove acetyl and acyl (fatty acid) groups from lysine residues in many proteins, including transcription factors, histones, and others. SIRT2-mediated deacetylation leads to changes in chromatin structure, gene regulation, intracellular microtubular network organization, and other effects. In the context of β cells, NAD^+^-dependent SIRT2 function serves as a feedback mechanism that controls β cell proliferation. During glycolysis, conversion of glucose to pyruvate requires reduction of 2 NAD^+^ molecules to NADH, depleting NAD^+^ and limiting SIRT2 activity. This NAD^+^ dependency places SIRT2 within a broader class of redox-sensitive sirtuins that act as metabolic sensors, integrating nutrient availability with regulatory functions in mitochondrial metabolism and cellular growth (13). Conversely, in a low-glucose, nonglycolytic environment, NAD^+^ is available and SIRT2 is active as a deacetylase, limiting the proliferation of β cells. The highlighted work by Wortham et al. now reveals that in a high-glucose environment, glucose serves as an indirect and modest metabolic SIRT2 inhibitor, and these events are associated with induction of mouse and human β cell proliferation.

How did Wortham and colleagues elect to focus on SIRT2? It is well known and widely documented that juvenile human and rodent β cells proliferate in response to glucose stimulation. Conversely, it is equally well known and documented that aged human and rodent β cells fail to proliferate in response to glucose. In this study, the authors compared their previously generated proteomics data sets from juvenile and adult mouse islets (14) and identified SIRT2 as a protein that was abundant in adult β cells and reduced in juvenile β cells. They then performed a series of genetic and pharmacologic studies exploring the role of SIRT2 in mouse and human β cell proliferation.

In both human and mouse ex vivo islets, regardless of the subject’s age, inhibiting or genetically silencing SIRT2 unleashed a proliferative response in β cells. Intriguingly, while human β cells proliferated in response to SIRT2 inhibition in both physiological and hyperglycemic conditions, mouse β cells only proliferated under hyperglycemic conditions. The major SIRT2 deacetylation target proteins identified were mitochondrial oxidative phosphorylation (OxPhos) regulatory proteins. Loss of acetylation of these proteins was associated with β cell proliferation. Wortham et al. recapitulated these events in vivo by treating mice with small-molecule SIRT2 inhibitor drugs and SIRT2 antisense oligonucleotides (ASOs) targeted to the β cell through conjugation to the GLP1-derived peptide exendin 4. In mice, systemic treatment with SIRT2 inhibitors promoted adaptive proliferation only in hyperglycemic states, without losing the braking effect of the deacetylating SIRT2 activity once glucose returned to normal levels. These interesting findings suggest that SIRT2 inhibitors may have potential use as human β cell–regenerative drugs.

## Looking into the future of SIRT2 inhibitor development

Like all newly proposed drug classes, these findings must pass the test of time through drug development, but if they do, the implications could be notable. The most impactful outcome may be that the β cell–regenerative drug field gains a new member that could be targeted exclusively to β cells. Notably, drug-induced hypoglycemia commonly occurs with insulin and is a theoretical risk associated with other classes of β cell–regenerative drugs. SIRT2-targeted approaches could be active in β cells only when blood glucose is elevated, but not when glucose levels are normal or low. Wortham et al. argue that this feature might be helpful in preventing hypoglycemia. SIRT2-based therapies could be envisioned as an orally delivered small molecule based on existing SIRT2 selective inhibitors AGK2 and AK-1, Sir-Real structures, or as an injectable, exendin-linked ASO.

This provocative line of work is in its early phases, and like all good studies, the findings of Wortham et al. raise new questions. First, SIRT2 inhibitors seem to allow reacetylation of mitochondrial OxPhos proteins, and this is apparently beneficial for β cell function and enhanced insulin secretion. But how do SIRT2 inhibitors drive β cell proliferation? Although proliferation is typically associated with changes in cell-cycle activators (cyclins, cyclin-dependent kinases, etc.), analogous changes were not observed in this study, so we have no insight into the pathway through which SIRT2 inhibitors lead to cell-cycle activation in β cells. Second, if SIRT2 is a key deacetylase that maintains β cell–cycle quiescence, what are the corresponding acetylases that reacetylate SIRT2 target proteins during proliferation? Third, why and how do human β cells, but not their mouse counterparts, proliferate at physiological glucose concentrations in response to SIRT2 inhibition, and how could the potential for inappropriate β cell mass expansion in SIRT2-treated humans be managed? A fourth question relates to the need for β cell drug targeting with exendin 4. As the authors note, GLP receptors are present in many tissues, including β cells, multiple brain regions, heart, and elsewhere. Will GLP1-targeted drugs have undesirable off-target effects? Our group’s studies of harmine have yielded no evidence of its off-target effects despite the wide distribution of its target kinase, DYRK1A, but this may or may not apply with GLP1-targeted ASOs or other drugs. Finally, the authors note that glucose makes β cells perform “work,” i.e., glucose forces β cells to increase insulin gene transcription, insulin synthesis, posttranslational processing, trafficking through the Golgi and ER, packaging in dense-core secretory granules, and, ultimately tightly regulated and energy-requiring secretion. This increased demand is referred to as “β cell work,” a series of energy-intensive processes of insulin production, including transcription, translation, and processing in the ER and Golgi. But the identity of the “β cell work sensor” remains unknown, as does SIRT2’s role in this system.

Some of these issues are nicely addressed in the limitations section of Wortham et al.’s Discussion. Other future questions that are likely on the authors’ minds are: (a) Might SIRT2 inhibitors synergize with other classes of potential β cell–regenerative drugs? (b) SIRT2 inhibition may be helpful for glucotoxicity or glucose-induced β cell work, but will it be helpful in the twin common menace in T2D: combined gluco-lipotoxic stress? We look forward to seeing how this unanticipated story unfolds in the next months and years.

## Version 1 10/01/2025

Electronic publication
